# Treatment and outcome of patients with metastatic NSCLC: a retrospective institution analysis of 493 patients

**DOI:** 10.1186/1465-9921-14-139

**Published:** 2013-12-18

**Authors:** Niels Reinmuth, Nadine Payer, Thomas Muley, Hans Hoffmann, Felix JF Herth, Matthias Villalobos, Michael Thomas

**Affiliations:** 1 Department of Thoracic Oncology, Thoraxklinik at the University of Heidelberg, 69126 Heidelberg, Germany; 2 Department of Thoracic Surgery, Thoraxklinik at the University of Heidelberg, 69126 Heidelberg, Germany; 3 Department of Pneumology & Respiratory Critical Care Medicine, Thoraxklinik at the University of Heidelberg, 69126 Heidelberg, Germany; 4 Translational Lung Research Center Heidelberg, German Center for Lung Research, Heidelberg, Germany; 5 Department of Thoracic Oncology, Lung Clinic Grosshansdorf, Wöhrendamm 80, D-22927 Grosshansdorf, Germany

**Keywords:** NSCLC, Chemotherapy, Therapy lines, Predictive factors, Palliative therapy

## Abstract

**Background:**

Most patients with metastatic non-small cell lung cancer (NSCLC) will face treatment with systemic therapy. Current clinical studies are demonstrating improvements in chemotherapy and overall survival. However, it remains unclear whether these results are translated into clinical practice.

**Methods:**

We reviewed all stage IV NSCLC patients without second malignancies that were diagnosed from 2004 to 2006 at our institution. 493 consecutive patients were included into this retrospective analysis and were followed-up until end of 2011.

**Results:**

352 patients (71.4%) received systemic therapy for up to 7 lines. For most patients, adjustments of dosages or applications had to be made at some point of the treatment, but the total applied dose remained generally close to the intended dose. The best disease control (BDC) rate decreased with increasing therapy lines from 59.7% to about 35%. Patients with palliative local therapy but no systemic treatment demonstrated inferior survival (median 2.9 versus 8.7 months, p < 0.001). The median interval between last treatment and death was 50 days and 15 days for chemotherapy and anti-EGFR therapy, respectively. BDC to the previous therapy lines was predictive for improved BDC to third- but not second-line therapy. Performing multivariate analysis, BDC to previous therapy, never-/ former-smoking status, and age > 70 years were associated with improved survival performing third-line therapy.

**Conclusions:**

Stage IV NSCLC patients may receive substantial systemic therapy resulting in response and median survival rates that are comparable to data from clinical studies. However, preselection factors are increasingly important to improve therapy outcome and life quality.

## Background

Lung cancer remains the leading cause of cancer-related deaths in the Western civilization with a median survival of only 8 months for patients with stage IV non-small cell lung cancer (NSCLC) treated with platinum-based therapy [1,2]. In recent years, new developments in systemic therapies have yielded extended survival, at least in large phase III trials [3]. For example, the preselection of NSCLC patients harboring EGFR mutations identified a subgroup with improved response to both EGFR tyrosine kinase inhibitors and chemotherapy. Also, the addition of Bevacizumab to chemotherapy has led to favorable response rates and extended overall survival [4]. Taking these advances together, prolonged survival rates have been described in large phase III studies compared to earlier data [2]. However, it remains speculative whether highly selective patient collectives as they are recruited to clinical phase III studies represent the general population of lung cancer patients in the daily routine, and whether the outcome is comparable to data from clinical studies [5]. For example, in a Veterans Affairs Central Cancer Registry containing 20,511 NSCLC cancer patients from 2003 to 2008, guideline-recommended chemotherapy treatment was received only by 34% of all metastatic patients aged between 65 and 74 years [6]. This percentage was even lower with increasing patient age. Moreover, the benefit of chemotherapy beyond second-line treatment has only been marginally addressed in clinical studies. Still, an increasing subset of patients will receive more than two therapy lines [7].

Since most metastatic NSCLC patients will inevitably die of their disease, quality of life and integration of palliative care in the management of advanced cancers have recently gained much attention. In a landmark study, Temel and coworkers demonstrated a survival benefit for patients assigned to early palliative care versus those in the standard care group [8]. Interestingly, both groups had comparable numbers of chemotherapy regimens while patients assigned to early palliative care had a significantly longer time between their last infusion dose and death [9]. However, data on intervals between last chemotherapy and death from clinical practice is missing.

To address these questions, we reviewed metastatic NSCLC patients diagnosed and treated in our institution within a defined time interval with emphasis on delivered chemotherapy treatment and potential prognostic factors to identify patients most likely benefiting from subsequent lines of systemic therapy.

## Patients and methods

Using the hospital information system and medical records, we retrospectively reviewed all lung cancer patients who were diagnosed at our institution between January 1st, 2004, and December 31st, 2006, and consented in writing for analyses of their data. Patients with further malignancies including second primary lung cancers were excluded to avoid any bias due to different outcome [10]. Upon approval by the local Ethic Committee, patients and their treating physicians were contacted, and the follow-up statuses were completed (S-612/2012). NSCLC staging was performed according to both the 6th and, retrospectively, the 7th edition of the UICC criteria. The smoking status was assessed at diagnosis of the lung cancer. Patients with total consumption of less than 100 cigarettes were classified as neversmokers.

### Systemic therapy

For each therapy line, the duration of therapy was calculated from the first to the last application day. The applied dose compared to the intended dose was calculated for each cycle and each therapy line. Delays of continuation of systemic therapy of 3 or more days due to decelerated recovery or concurrent medical problems such as infections were noted. Best response that was achieved at each therapy line was assessed according to RECIST (v1.1) performing CT scans (usually every 2 cycles during therapy), and the best disease control rate (BDCR) was calculated [11]. In general, application of chemotherapy was repeated every 3 weeks. For treatment with EGFR Tyrosine kinase inhibitors (TKI), the duration of a therapy cycle was defined as 4 weeks. All patients entered a follow-up program with 3-monthly visits for the first 2 years, 6-monthly visits after 2-5 years and yearly visits thereafter [12]. All follow-up visits included physical examination, lung function tests and a chest radiograph.

### Statistical analysis

We scheduled December 31st, 2011 as the census date. Survival was defined as interval between date of diagnosis and death if not stated otherwise. Because of the nature of this disease and the inability to ensure accuracy and consistency across all patients due to the retrospective character of this study, cause of death was not captured but identified by clinical means. Survival analysis was assessed using the Kaplan Meier method. Univariate analyses were done performing log rank regression tests. Throughout, a p-value of <0.05 was considered to be statistically significant. Multivariate analyses were performed using stepwise multiple Cox regression (entry p = 0.05, exit p = 0.10).

## Results

**Figure 1:**
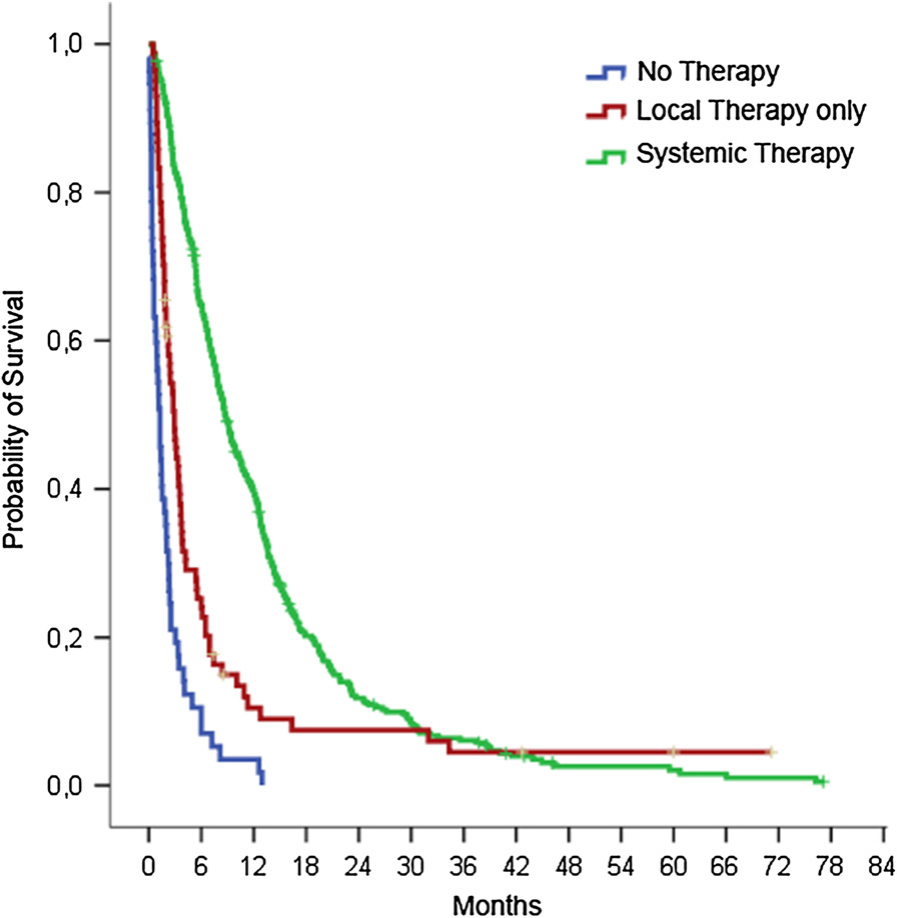
**Overall survival of metastatic NSCLC patients.** Patients received either systemic therapy, palliative local treatment without systemic therapy or no anti-cancer treatment. Median survival rates were 8.7, 2.9, and 1.3 months respectively (p < 0.001).

A total of 493 patients with stage IV NSCLC were identified (Table 1). 57 (11.6%) patients (median age 72 years) did not receive any anti-cancer treatment due to poor performance status or upon patient denial. These patients were characterized by a median survival of 1.3 months (95%-CI 0.7-1.5 months). 352 patients (71.4%) received at least one line of systemic treatment (Table 2). 84 patients (17.0%) underwent only local treatment by surgery or radiotherapy but had no systemic therapy due to the above mentioned reasons. From diagnosis, these patients had a median overall survival of 2.9 months (95%-CI 2.3-3.6 months) that was significantly inferior to patients treated with systemic therapy (median 8.7 months; 95%-CI 7.6-9.8 months; p < 0.001; Figure 1).

**Table 1:** Baseline characteristics of the patients

	**No therapy (n = 57)**		**Patients receiving any therapie (n = 436)**		**Patients with systemic therapy (n = 352)**		**Patients with local therapy only (n = 84)**	
**Age at diagnosis (years)**								
Median	72		62		62		65	
Range	50-84		34-86		34-86		44-85	
< 70 years	22	(38.6)	321	(73.6)	261	(74.1)	60	(71.4)
≥ 70 years	35	(61.4)	115	(26.4)	91	(25.9)	24	(28.6)
**Sex**								
Male	46	(80.7)	299	(68.6)	237	(67.3)	62	(73.8)
Female	11	(19.3)	137	(31.4)	115	(32.7)	22	(26.2)
**Smoking status**								
Neversmoker	2	(4.8)	35	(9.9)	35	(12.5)	0	(0)
Former smoker	25	(59.5)	143	(40.5)	115	(40.9)	28	(38.9)
Current smoker	15	(35.7)	175	(49.6)	131	(46.6)	44	(61.1)
Unknown	15		83		71		12	
**Stage**								
Stage IV, UICC 6. edition	57	100	436	(100)	352	(100)	84	(100)
Stage IV, UICC 7. edition	56	(98.2)	419	(96.1)	338	(96.0)	81	(96.4)
Stage IIIB, UICC 7. edition	1	(1.8)	17	(3.9)	14	( 4.0)	3	(3.6)
**Histology**								
Adenocarcinoma	25	(43.9)	255	(58.5)	222	(63.1)	33	(39.3)
Squamous cell carcinoma	18	(31.6)	85	(19.5)	56	(15.9)	29	(34.5)
Large cell undifferentiated carcinoma	14	(24.6)	96	(22.0)	74	(21.0)	22	(26.2)

Percentages are displayed in brackets. Stage classification according to either 6th or 7th UICC edition. Because of rounding, not all percentages sum to 100.

**Table 2:** Therapy of all patients reflected in the present analysis

**Therapy modality**	**n**	**%**
*Patients receiving systemic therapy*	352	71.4
Only Systemic therapy	122	24.7
Systemic therapy and radiotherapy	183	37.1
Systemic therapy and surgery	17	3.4
All therapy modalities	30	6.1
*Patients receiving only local therapy*	84	17.0
Only radiotherapy	67	13.6
Only surgery	5	1.0
Surgery and radiotherapy	12	2.5
*Patients receiving no anti-cancer treatment*	57	11.6

### Systemic therapy

**Figure 2:**
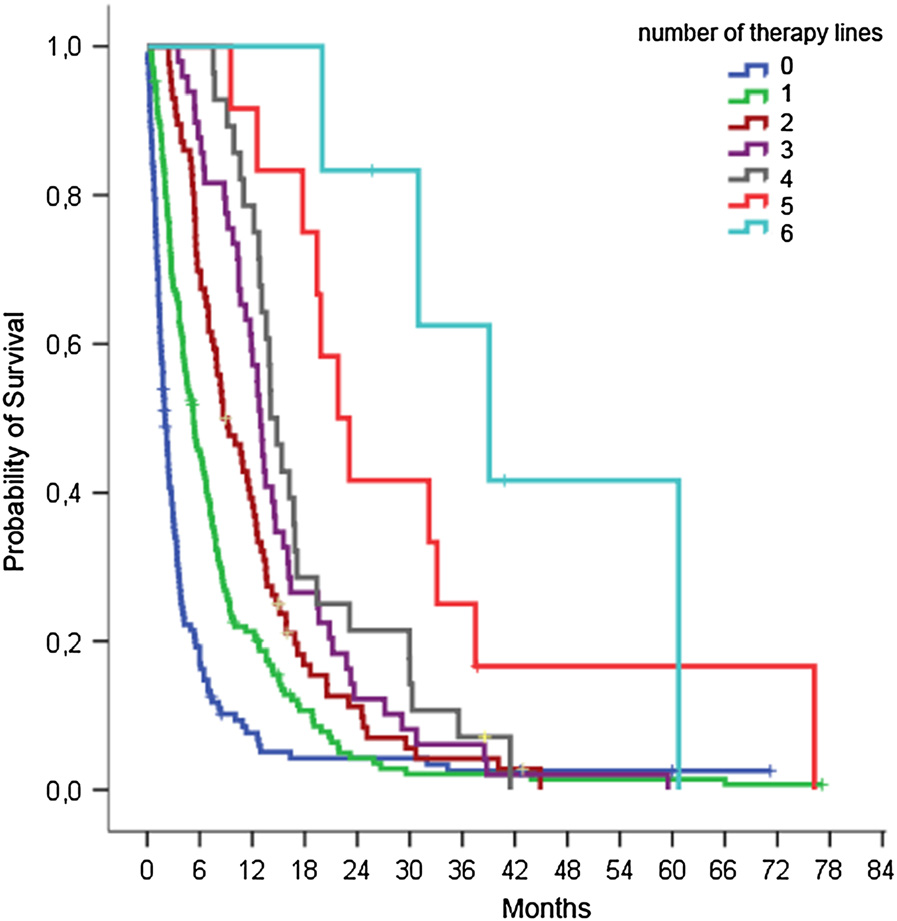
**Overall survival according to applied lines of systemic therapy.** Survival rates were calculated from date of diagnosis. Median survival significantly improved with increasing numbers of lines of systemic therapy (p < 0.001). Patients with 0–6 lines of systemic treatment had a median survival of 2.0, 5.3, 8.7, 13.0, 14.1, 21.8, and 39.1 months, respectively.

352 patients with stage IV NSCLC received systemic therapy which was given for up to 7 lines. The median age of patients with systemic therapy was 62 years with 25.9% of 70 years or older. Cumulative numbers of therapy lines were associated with improved survival but with decreasing BDCRs (Table 3, Figure 2). Interestingly, even in higher therapy lines, a BDCR of around 35% could be observed. Using the 7th edition of UICC criteria, 14 patients would be reclassified as having stage IIIB disease which did not significantly alter the study results. In addition to systemic therapy, a total of 213 patients (60.5%) received palliative radiotherapy mainly due to symptomatic cerebral or bone metastases. 38 patients (10.8%) received at least one surgical procedure with palliative intention including 17 resections of metastases (brain n = 5, skin n = 7, rip n = 2, cervical lymph nodes n = 3), 12 pleurectomies, and 2 surgical drainages of pleural empyema. For 9 patients undergoing palliative lung tumor resection (3 pneumonectomies, 1 bilobectomy, 5 lobectomies), a median survival of 15.6 months (95%-CI 0–12.3 months) was noted.

**Table 3:** Application, response and survival of systemic therapy

	**Patients**		**Response**				**Overall survival**	
**Lines of systemic therapy**	**n**	**%**	PR*	SD*	NE*	PD*	**Median OS**	**95% CI**
1	352	100	30.7	29.0	20.7	19.6	7.6	6.8-8.5
2	183	52.0	14.8	31.7	19.7	33.8	6.2	5.0-7.4
3	97	27.6	12.4	23.7	22.7	41.2	5.2	3.5-7.0
4	48	13.6	14.6	20.8	31.3	33.4	5.1	3.7-6.5
5	20	5.7	5.0	30.0	10	55.0	4.5	1.9-7.1
6	8	2.3	37.5	12.5	12.5	37.5	11.1	3.1-19.1
7	2	0.6	0	0	50	50		

Survival is stated in months from the beginning of the respective line of systemic therapy. Response was assessed according to RECIST: PR, Partial response; SD, stable disease; NE, not evaluable; PD, progressive disease. CI, confidence interval. OS, overall survival. * Because of rounding, not all percentages sum to 100.

As first-line therapy, 276 patients (78%) received platinum-based combination therapy with gemcitabine as the most frequent combination partner. For 145 patients (which were younger than 70 years in 93.8%), cisplatin was used. Due to toxicity, this treatment combination had to be switched to carboplatin or continued as monotherapy in 43 and 2 cases, respectively. Patients with initiation of cisplatin based treatments had a similar BDCR (PR 40% SD 32%) and overall survival (9.6 months; 95%-CI 7.8-11.4 months) compared to patients treated with carboplatin based combinations (PR 32%, SD 28%; median survival 10.5 months; 95%-CI 8.6-12.3 months). In contrast, patients treated with monotherapy had an inferior BDCR (PR 9%, SD 25%) and a survival of 4.4 months (95%-CI 2.5-6.2 months; p < 0.001). Of 248 patients with platinum based first-line chemotherapy not entering clinical studies, 157 patients (63.3%) experienced a dose reduction due to toxicity. However, the applied mean dosage of all patients compared to the intended dosage was only moderately reduced (Table 3). In contrast, first-line monotherapy could be given only for a mean of 2.2 cycles and with an intended dosage of 83%.

Subsequent therapy was applied largely as monotherapy with docetaxel, pemetrexed, erlotinib and vinorelbine as most frequent drugs. Similar to first-line therapy, the application of subsequent lines had to be modified only to a minor extent by delaying or reducing the dosage (Table 4). Most frequent reasons for terminating the current therapy were disease progression and worsening of health status. The median treatment duration with EGFR TKI was 8 weeks (2–86 weeks) for second-line and 6 weeks (2–78 weeks) for third-line treatment, respectively. Of note, the response to second-line treatment with erlotinib (PR 15%, SD 30%) was comparable to docetaxel (PR12%, SD 29%). The median interval between last chemotherapy and death was 50 days (266 patients; 95%-CI 40–60 days) while the median time between stopping anti-EGFR therapy and death was 15 days (68 patients; 95%-CI 10–20 days).

**Table 4:** Applied dosages and dose modifications of chemotherapy

**Treatment and patients**	**Mean dosage**	**Mean number of cycles**	**Patients with dose reduction**	**Patients with delays**	**Mean delay (range)**	**Break-off current therapy line**
	**%**	**N**	**N (%)**	**N**	**Days**	**%**
**1st line**						
Platinum (n = 248)	96.1	3.9	47 (19.0)*	101	15 (3–52)	57.7
For combination therapy: combined cytotoxic drug (n = 248)	84.1	3.9	146 (58.9)*	101	15 (3–52)	57.7
Monotherapy (n = 60)	82.9	2.2	31 (51.7)	13	14 (7–28)	91.6
**2nd line**						
Monotherapy (n = 117)	95.4	3.2	20 (17.0)	26	15 (4–49)	77.7
EGFR-TKI (n = 41)	**	4.4	**	**	**	NA
Platinum-basedcombination (n = 18)	NA	3.6	14 (77.8)	8	18 (4–31)	55.6
**3rd line**						
Monotherapy (n = 49)	95.2	2.9	8 (16.3)	9	12 (7–24)	81.6
EGFR-TKI (n = 36)	**	3.4	**	**	**	NA
Platinum-basedcombination (n = 7)	NA	3.3	4 (57.1)	3	12 (7–21)	71.4

Patients who entered clinical studies were excluded. EGFR TKI, tyrosine kinase inhibitor targeting the epidermal growth factor receptor. NA, not applicable.

*36 patients (14.5%) received a dose reduction of platinum and combined cytotoxic drug.

**no reliable data available.

### Predictive and prognostic factors of second- and third-line therapy

A disease control as BDCR at previous systemic therapy was predictive for a higher BDCR at third-line treatment (Odds Ratio = 0.08; 95% CI 0.01-0.7; p = 0.047) but not for improved BDCR at second-line therapy (p > 0.05). Moreover, age of 70 years or older was associated with improved BDCR (Odds Ratio = 0.27; 95% CI 0.11-0.68; p = 0.005) at second-line therapy but not at third-line therapy.

After univariate analyses, prognostic factors for overall survival from the initiation of second- and third-line therapy are displayed in Table 5. For example, a history of disease control at previous systemic therapy significantly correlated with favorable overall survival from second- and third-line therapy. Similarly, patients with progressive disease during or within 9 months of initiation of first-line platinum-based chemotherapy had a significantly worse survival after initiation of second-line treatment (p = 0.003 and p = 0.005, respectively; data not shown) which was particularly true for patients with adenocarcinoma (p = 0.05 and p = 0.032, respectively). Performing multivariate testing, only age and tumor control at previous therapy lines remained significant prognostic factors for both second- and third-line therapy (Table 6).

**Table 5:** Univariate analysis of prognostic survival factors for 2nd and 3rd line therapy

	**Survival after 2nd line therapy**			**Survival after 3rd line therapy**		
	**HR**	**95%-CI**	**p-value**	**HR**	**95%-CI**	**p-value**
**Sex**			0.120			0.415
Female	0.77	0.56-1.07		0.83	0.53-1.30	
Male	1			1		
**Age**			0.015			0.007
< 70 years	1			1		
≥ 70 years	0.63	0.43-0.91		0.48	0.28-0.82	
Smoking status*			0.032			0.012
Never smoker	0.56	0.34-0.93		0.43	0.21-0.86	
Former smoker	0.69	0.48-0.99		0.53	0.32-0.87	
Current smoker	1			1		
**Histology**			0.037			0.597
Adenocarcinoma	0.59	0.40-0.88		0.89	0.46-1.7	
Squamous cell carcinoma	0.64	0.38-1.09		1.19	0.53-2.63	
Large cellundifferentiated carcinoma	1			1		
**Response to previous therapy line**			0.001			< 0.001
Disease control	0.58	0.41-0.81		0.44	0.29-0.69	
Progress	1			1		
**Response to both previous therapy lines**			NA			< 0.001
Disease control in both previous lines	─	─		0.26	0.13-0.51	
Disease control in any previous line	─	─		0.53	0.28-0.99	
Progress in both previous lines	─	─		1		

HR, hazard ratio; CI, confidence interval. *Patients with known smoking status.

“─” indicates that data could not be calculated.

**Table 6:** Multivariate analysis of prognostic survival factors for 2nd and 3rd line therapy

	**Survival after 2nd line therapy**			**Survival after 3rd line therapy**		
	**HR**	**95% CI**	**p-value**	**HR**	**95% CI**	**p-value**
*Age*			0.011			0.034
< 70 years	1			1		
≥ 70 years	0.61	0.41-0.89		0.54	0.31-0.95	
*Smoking status*			>0.1			0.033
Never smoker	─	─		0.35	0.17-0.74	
Former smoker	─	─		0.59	0.35-0.98	
Current smoker	─	─		1		
Unknown	─	─		0.72	0.38-1.36	
*Histology*			0.076			>0.1
Adenocarcinoma	0.64	0.42-0.96		─	─	
Squamous cell carcinoma	0.61	0.35-1.05		─	─	
Large cell undifferentiated carcinoma	1			─		
*Response to previous therapy line*			0.003			>0.1
Disease control	0.60	0.42-0.84		─	─	
Progress	1			─		
*Response to both previous therapy lines*			NA			< 0.001
Disease control in both previous lines	─	─		0.29	0.14-0.59	
Disease control in any previous line	─	─		0.73	0.38-1.40	
Progress in both previous lines	─	─		1		

HR, hazard ratio; CI, confidence interval.

“─” indicates that data could not be calculated.

### Complete resection of stage IV NSCLC disease

Tumor resection with curative intention was performed on 16 patients including 7 pneumonectomies, 1 bilobectomy and 8 lobectomies. In addition, 9 and 7 patients underwent resection of pulmonary and cerebral metastases, respectively. Moreover, 11 patients received radiotherapy of the thorax (n = 4) and/or cerebrum (n = 9). Deploying the new TNM stage classification, 2 patients with ipsilateral pulmonary metastases would be reclassified as stage IIIB. All 16 patients with curative intended surgery had a median survival of 13.6 months (95%-CI 0–33.2 months) with 2 patients being still alive after 5 years. Systemic therapy was applied to 9 patients who had a median survival of 19.9 months (95%-CI 4.6-35.2).

## Discussion

In recent years, few data on single institution experiences regarding chemotherapy treatment of NSCLC patients has been published, with the current analysis being one of the largest collectives [13,14]. In general, results from large phase III trials seem to be reproducible in the clinical routine. With a response rate of 37% for first-line platinum-containing regimens, our analysis demonstrated even improved results compared to a large phase III study analyzing four chemotherapy regimens (response rate 19%) [2] and some newer studies (response rates of 28.2% and 21.6% for cisplatin/gemcitabine combinations, respectively) [15,16]. Moreover, the median overall survival of our collective receiving first-line platinum-based chemotherapy was somewhat comparable to these studies reporting survival data between 7.8 months [2] and 13.1 months [16]. As reported in these studies, patients seemed to have recieved subsequent therapy to a comparable extent as in our study [16,17]. Complete surgical resection of stage IV disease may be an option for highly selected patients with stage IV disease [18,19]. However, the patient number in our study was too small to draw any conclusions.

Similar to others [7,13], our data shows a decreasing BDCR with progressing therapy-lines. Again, for second-line therapy, our BDCR and survival rates somewhat resemble data from clinical studies regarding pemetrexed, docetaxel, and erlotinib [17,20,21]. However, since we could not retrospectively assess the performance status we cannot exclude a possible influence of a poor performance status on the outcome of advanced therapy lines. In our study, all drugs used for therapy beyond first-line treatment showed comparable outcome. For example, the response rate of erlotinib was similar to docetaxel (15% versus 12%). Again, this finding is in congruence with data from previous studies with unselected patients regarding the EGFR mutational status [17,22]. Few clinical studies focusing on third-line therapy of NSCLC patients have been conducted [20,21]. Importantly, the overall survival rates after initiation of advanced therapy lines were superior than the reported outcome of patients with best supportive care alone after failure of first-line or second-line chemotherapy [20,21].

The response to previous therapies may be both predictive and prognostic for subsequent therapies. Due to small patient numbers we analyzed BDCR rather than response showing inconsistent results regarding its predictive value for response in subsequent therapy lines which is similar to previous studies [7,13,14]. Disease control with previous therapies has been repetitively associated with improved survival for subsequent chemotherapy which has been also demonstrated in our analysis [7,13,23]. Moreover, patients with progressive disease upon first-line chemotherapy have been associated with poor prognosis in previous studies with overall survival times around 5 months and poor responses to subsequent therapy [24,25]. Interestingly, in a recent phase III trial, patients with stage IV adenocarcinoma and progressive disease within 9 months of induction of first-line chemotherapy showed a significant survival benefit with the addition of a multi-tyrosine kinase inhibitor to second-line therapy with docetaxel [26]. As analyzed for the first time in the current study, this group was characterized by a significant worse prognosis. Finally, patients aged 70 years or more showed improved BDCR and overall survival for second-line therapy. Interestingly, similar data has been reported for first-line therapies [27]. However, several other studies do not support this finding [7,28] which may possibly be related to high patient selection. Collectively, patients with advanced NSCLC may most likely benefit from advanced therapy lines when they experienced disease control after first- and second-line treatments and have a favorable performance status [7].

Similar to other experiences, 50% of patients diagnosed with metastatic NSCLC received more than 1 line of systemic therapy and 30% of all patients were treated with 3 or more treatment lines [13,29]. Dose reductions and delays of chemotherapy were a frequent necessity. However, in the majority of patients, this led only to a modest reduction of the total applied dosage and minor delays. In our analysis, the number of cycles was slightly lower as observed in some clinical studies, likely due to higher selection of patients entering clinical studies [15,17,20].

Patients that could not receive systemic therapy were characterized by a much reduced median survival of 1.3 months which was even worse compared to earlier clinical studies randomizing patients for best supportive care only [5,30]. In contrast, patients treated with first-line monochemotherapy had a slightly improved survival which was still significantly lower compared to patients treated with platinum-based combinations. Hence, while we were not able to directly address the influence of comorbidities in our results, we agree that the health performance status is a major prognostic factor regarding survival and treatment options for patients with metastatic NSCLC [31-33].

Importantly, the limited administration of systemic therapy within the last months of the patient’s life has been accepted as one parameter of measuring quality of life [34]. In a clinical study, patients with metastatic NSCLC treated with systemic therapy and early palliative care had a significantly longer interval between last chemotherapy dose and death than patients treated with standard oncology care alone (median 64 vs 40.5 days; p = 0.02). Interestingly, the former patients were characterized by a significantly longer overall survival [8,9]. With a median of 50 days in our study, the interval between termination of systemic treatment and death was somewhat longer compared to patients treated with standard oncology care alone in this study [9] and comparable to other data [29]. Identifying the appropriate time for ending systemic therapy and intensifying palliative care remain important challenges for improving oncologic treatment for our patients.

In summary, results from clinical studies are translated into the clinical practice leading to increasing therapy lines and advanced survival. Moreover, our analysis underlines the importance of preselecting patients. Identification of both predictive and prognostic factors as well as the best time point of assessing these factors will be critical to select patients who will most likely benefit from intensified systemic therapy.

## Competing interests

N. Reinmuth works as a consultant for Roche, Lilly, Amgen and received honoraria from Roche, Lilly, Novartis, Boehringer-Ingelheim. F.J.F. Herth worked as a consultant for Roche, Olympus, Uptake Medical, PneumRx, Aeris, Pulmonx, IPS, Astra Zeneca and received honoraria from Roche, Olympus, Uptake Medical, PneumRx, Aeris, Pulmonx, IPS, Astra Zeneca, Bayer Pharma, Boehringer Ingelheim, Lily, Pierre Fabre, Nycomed, Pfizer and Novartis. All other authors stated no potential conflicts of interest with any companies/organizations whose products or services may be discussed in this article.

## Authors’ contributions

All authors contributed substantially to conception, design, acquisition of data, analysis of data and interpretation of data. All authors were involved in drafting or revising the manuscript and approved the final version.
